# Recombinant Alkaline Phosphatase Prevents Acute on Chronic Liver Failure

**DOI:** 10.1038/s41598-019-57284-z

**Published:** 2020-01-15

**Authors:** Cornelius Engelmann, Danielle Adebayo, Marc Oria, Francesco De Chiara, Simone Novelli, Abeba Habtesion, Nathan Davies, Fausto Andreola, Rajiv Jalan

**Affiliations:** 1 Liver Failure Group, Institute for Liver and Digestive Health, University College London, Royal Free Campus, London, United Kingdom; 2 Section Hepatologie, Clinic for Gastroenterology and Rheumatology, University Hospital Leipzig, Leipzig, Germany; 3 Department of Mechanical and Aerospace Engineering, Sapienza University of Rome, Rome, Italy

**Keywords:** Gastroenterology, Liver, Liver cirrhosis

## Abstract

The lipopolysaccharide (LPS)– toll-like receptor-4 (TLR4) pathway plays an important role in liver failure. Recombinant alkaline phosphatase (recAP) deactivates LPS. The aim of this study was to determine whether recAP prevents the progression of acute and acute-on-chronic liver failure (ACLF). Eight groups of rats were studied 4-weeks after sham surgery or bile duct ligation and were injected with saline or LPS to mimic ACLF. Acute liver failure was induced with Galactosamine-LPS and in both models animals were treated with recAP prior to LPS administration. In the ACLF model, the severity of liver dysfunction and brain edema was attenuated by recAP, associated with reduction in cytokines, chemokines, liver cell death, and brain water. The activity of LPS was reduced by recAP. The treatment was not effective in acute liver failure. Hepatic TLR4 expression was reduced by recAP in ACLF but not acute liver failure. Increased sensitivity to endotoxins in cirrhosis is associated with upregulation of hepatic TLR4, which explains susceptibility to development of ACLF whereas acute liver failure is likely due to direct hepatoxicity. RecAP prevents multiple organ injury by reducing receptor expression and is a potential novel treatment option for prevention of ACLF but not acute liver failure.

## Introduction

Acute-on-chronic liver failure (ACLF) is a severe complication of chronic liver diseases characterized by acute decompensation resulting in multiorgan-failure and associated with systemic inflammation, increased liver cell death, and mortality^1–3^. Endotoxins, notably lipopolysaccharides (LPS), are pathogen associated molecular patterns (PAMPs)^2,4–6^, which have been shown in animal models of chronic liver disease to induce clinical and pathophysiological features typical of ACLF^7–9^. In alcoholic hepatitis elevation of circulating LPS was associated with increased risk of death and disease severity^10,11^. LPS initiates an inflammatory cascade by binding to Pattern Recognition Receptors (PRR), among which the Toll-like-receptors subtype 4 (TLR4) is the most important^12^. TLR4 is expressed on several cell types including immune cells, such as monocytes^13^ and parenchymal cells, such as hepatocytes^14,15^. The LPS-TLR4 axis is therefore a potential new therapeutic target in ACLF.

Sensitivity to LPS is enhanced in fibrotic liver. Although LPS may induce liver injury in individuals with healthy liver at high doses, tolerance of healthy hepatocytes to endotoxins is substantial and a full-blown hepatic failure due to infection is rare^16^. Acute liver failure (ALF) occurs in individuals with previously normal livers and is due to overwhelming liver injury and usually triggered by a hepatotoxic insult^17,18^. ACLF, in contrary, is often precipitated by infection^19^. These observations suggest that infection in general and the TLR4/LPS pathway may play a more important role in the pathogenesis of ACLF than ALF.

Although regarded as a biomarker of cholestasis, alkaline phosphatase (ALP) also plays an important anti-inflammatory role. Protective roles of intestinal alkaline phosphatase include the detoxification of free nucleotides and bacterial LPS^20^. It is thought to work through dephosphorylation of LPS reducing its activity^21^. Administration of recombinant alkaline phosphatase (recAP) has been shown to reduce inflammation in animal models and is being developed for the treatment of sepsis-related acute kidney injury (AKI)^22–24^. The aim of this study was to investigate the role of the LPS-TLR4 pathway in LPS-induced liver failure and determine whether recAP can prevent the development of ALF and ACLF in LPS-induced rodent models.

## Results

### Effect by recAP pre-treatment in ACLF

#### Clinical and hemodynamic effect of recAP pre-treatment

All animals were sacrificed 3 -hours after LPS or vehicle injection, respectively. Mean portal pressure (PP) was higher in the bile duct ligated (BDL) animals compared with the sham animals (p = 0.001) and did not change with recAP (Supplementary Fig. 2).

#### recAP reduces the severity of liver injury

**Figure 1:**
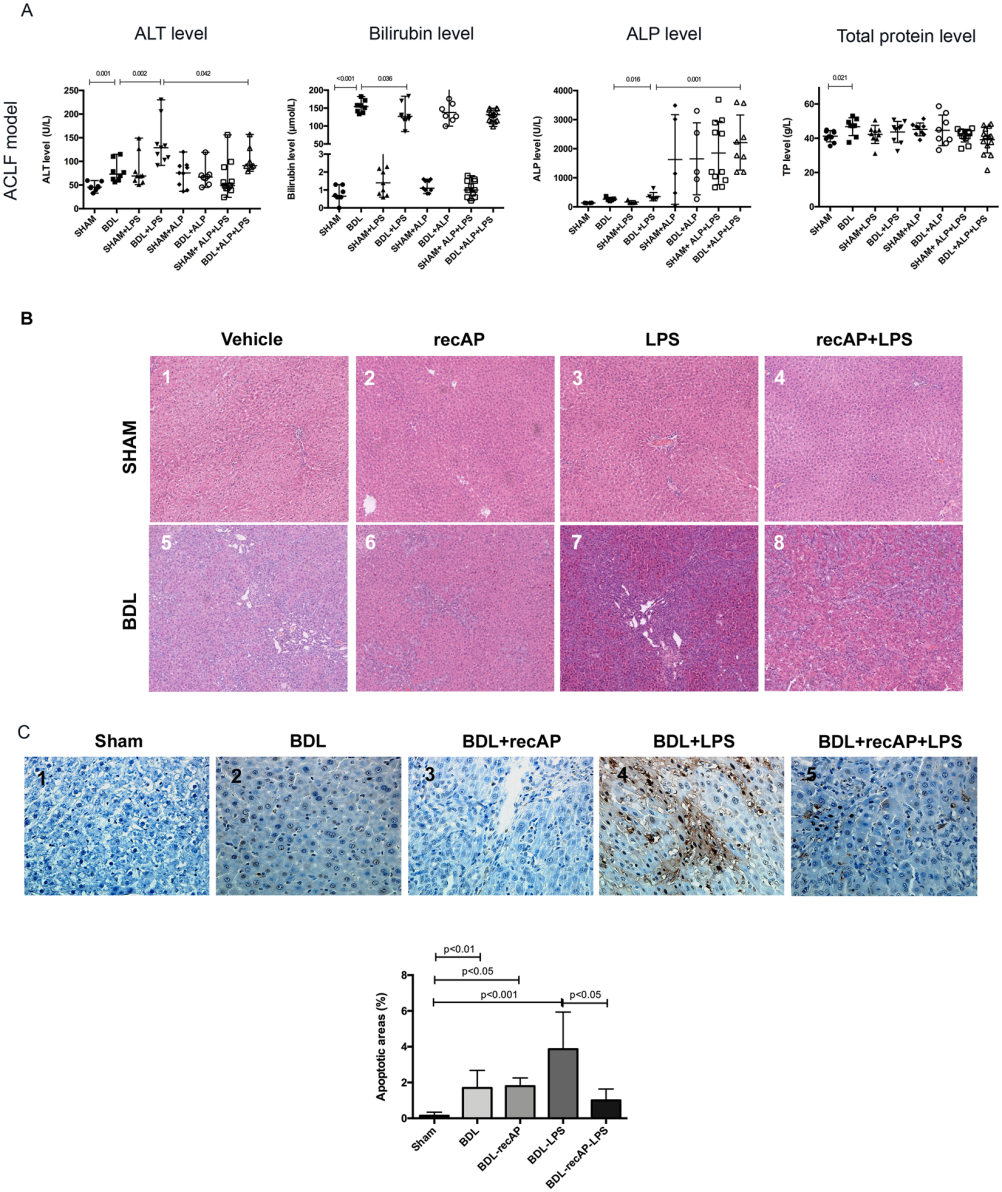
Liver biochemistry, H&E staining and apoptotic cell death (TUNEL staining) of liver tissue from the ACLF model. (**A**) Biochemistry parameters were measured in all animals per group. Only significant p-values were displayed in the graphs. Due to a high variance logarithmic scale was chosen to depict the results for ALT and Bilirubin. **(B)** Liver tissue from all groups of the ACLF model was stained with H&E (n = 4 per group). Normal histology was observed in the liver from the sham, sham + recAP animals (**B1** and **B2**, respectively). In the sham animals given LPS, there was evidence of increased inflammatory infiltrates (**B3**). Compared to the sham histology, the histology of the BDL animals (**B5**) showed evidence of ductal proliferation. Residual ductal proliferation was still observed in the liver from the BDL rats pre-treated with recAP; nevertheless, healthy residual hepatocytes could be observed (**B5–8**). When LPS was given to the BDL animals the liver histology showed evidence of necrosis and inflammatory cell infiltration **(B7).** Treatment with recAP in the BDL animals given LPS resulted in better preservation of hepatocytes **(B8)** (Magnification X10). **(C)** TUNEL staining of liver tissue was performed to detect apoptotic cell death in rats from the ACLF model (Magnification X40). RecAP pre-treatment markedly reduced apoptotic cell death in ACLF. TUNEL staining of liver slices showed a mild degree of apoptosis in the liver of the BDL and BDL + recAP animals **(C5 and C6)**. Livers from BDL + LPS animals presented with extensive apoptotic foci **(C7)**; the extent of the foci was markedly reduced in BDL + LPS animals pre-treated with recAP **(C8)**. Quantification of apoptotic areas was performed using ImageJ on images with 10x magnification (Sham n = 5, BDL + LPS n = 8; BDL, BDL + recAP n = 8, BDL + recAP + LPS n = 9). Group comparisons for continuous variables were performed by using Mann-Whitney U test. A p-value ≤ 0.05 was considered significant.

**Figure 2:**
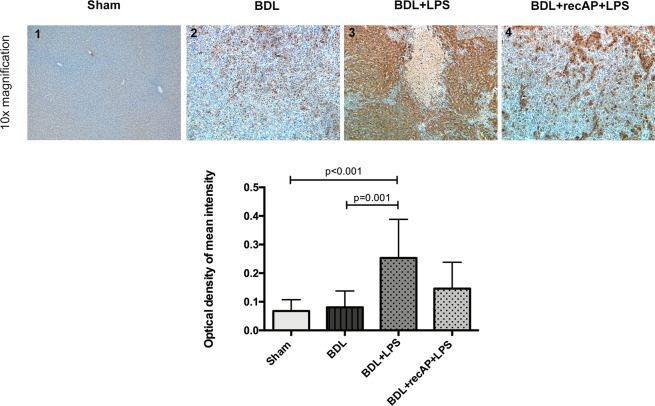
Effect of recAP on RIPK3 expression in liver tissue. Liver tissues of animals from the sham group (n = 4), BDL group (n = 2), BDL + LPS group (n = 5) and BDL + recAP + LPS (n = 5) were stained by immunohistochemistry for RIPK3. Whilst BDL animals showed a mild RIPK3 expression led LPS injection to a significant increase of RIPK3. Using recAP before LPS injection could abrogate the LPS effect on RIPK3 expression in the liver, although not reaching statistical significance. Quantification of DAB (RIPK3) intensity was performed by color deconvolution on ImageJ using a Fiji plugin. At 10x magnification five images per individual were taken and further analysed. Group comparison von performed by Kruskall-Wallis test. Only significant p-values were displayed in the figure.

Serum levels of alanine aminotransferase (ALT) (p = 0.001), bilirubin (p < 0.001) and ALP (p = 0.016) were significantly elevated in the BDL group compared to the sham-operated group. The BDL rats treated with LPS showed a further increase in serum ALT (p = 0.002) levels, which was significantly reduced in those pre-treated with recAP (p = 0.042). All recAP treated groups showed the highest levels of serum ALP demonstrating the presence of the drug (Fig. 1A, Supplementary Table 1). Histopathologically, recAP therapy had no significant impact on hepatic inflammatory cell infiltrates (Fig. 1B). Terminal deoxynucleotidyl transferase biotin-dUTP nick end labelling (TUNEL) staining showed no signs of apoptosis in sham and sham + recAP-treated animals (Supplementary Figs. 11.1 and 11.2). LPS treatment in sham animals induced apoptosis, which tend to be improved by recAP pre-treatment (Supplementary Figs. 11.3 and 11.4). Mild degree of apoptosis was also present in the liver of the BDL and BDL + recAP animals (Fig. 1C5,C6). Extensive apoptotic foci, shrunken nuclei and cytoplasmatic staining were found in livers from LPS-treated BDL animals (Fig. 1C7), which were significantly reduced by recAP (Fig. 1C8,E1). This TUNEL pattern suggested a different form of cell death after LPS injection, such as necroptosis. Immunohistochemistry of RIPK3, which is the mediator of necroptosis, confirmed the TUNEL results showing an increased expression after LPS injection in BDL animals which was numerically reduced by recAP (Fig. 2). Data therefore suggest, that chronic liver injury leads to a more pronounced liver injury in response to endotoxins and recAP is able to effectively abrogate this effect.

#### recAP treatment reduces hepatic and systemic cytokines and chemokines

**Figure 3:**
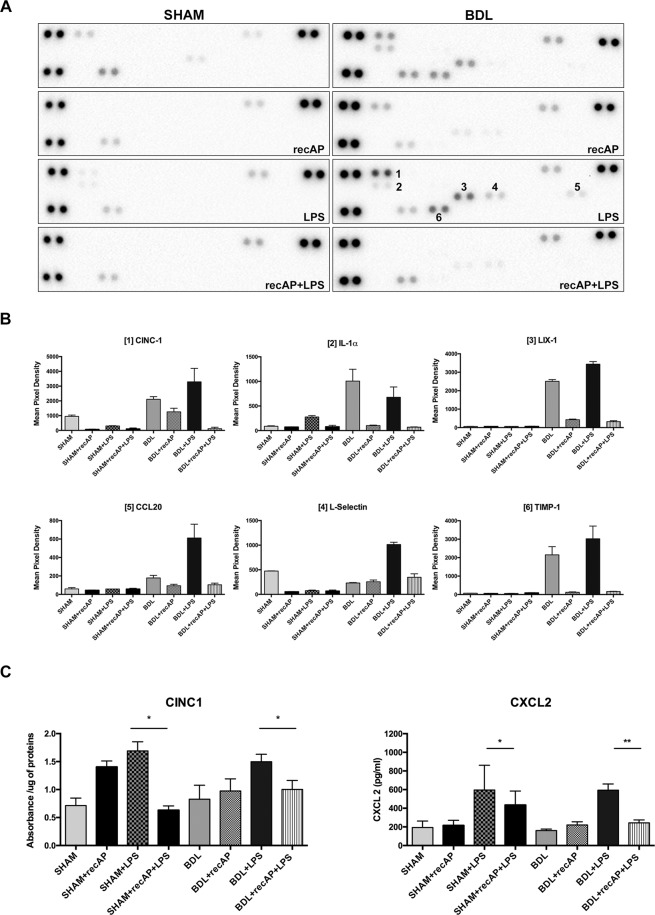
Cytokine expression profile in rats from the ACLF model. recAP treatment prevented upregulation of pro-inflammatory chemokines in the liver. Liver lysates of five individuals per group contributed to the pooled samples used to incubate the profiler membranes. **(A)** Up regulation of tissue metalloproteinase1 (TIMP1), lipopolysaccharide induced CXC chemokine (LIX), IL1a and L-Selectin was seen in the BDL group (top blot, right) compared to the sham controls (top blot, left). In the BDL + LPS group, besides further upregulation of tissue metalloproteinase-1 (TIMP-1) (dots number 6), LIX (dots number 3) and L-Selectin (dots number 4), a marked expression of CINC1 (dots number 1), IL1a (dots number 2) and CCL20 (dots number 5) was observed, compared to the BDL group. Pre-treatment with recAP resulted in a marked attenuation of the expression of these potent chemokines (second and bottom blots, right). The reference spots are those on the right and left edges of the blot. (**B**) Densitometric analysis of the proteins that were most modulated in the different treatment groups (mean pixel density ± SD). (**C**) recAP pre-treatment prevents upregulation of pro-inflammatory CINC1 and CXCL2 chemokines in the liver and plasma, respectively (**p < 0.01, *p < 0.05) when assessed by ELISA. Group comparisons for continuous variables were performed using Mann-Whitney U test. A p-value ≤ 0.05 was considered significant. Only significant p-values were displayed in the figure.

The rat proteome profiler showed upregulation of tissue metalloproteinase-1 (TIMP-1), CXC chemokine (LIX) and L-Selectin in the BDL + LPS group (Fig. 3A,B) in the liver. LIX is a murine neutrophil chemoattractant, and L-selectin is a cell adhesion molecule mediating lymphocyte capture and trafficking. In the BDL + LPS group, a more pronounced upregulation of TIMP-1, LIX and L-selectin was observed. In addition, over-expression of a cytokine-induced chemo-attractant, CINC-1, Interleukin-1a (IL1a), and CCL-20, a macrophage inflammatory protein was observed. Importantly, in the BDL + LPS rats pre-treated with recAP, a marked attenuation in the expression of these potent chemokines was demonstrated (Fig. 3B).

Hepatic expression of cytokine-induced neutrophil chemoattractant 1 (CINC-1) was confirmed by enzyme linked immunosorbent assay (ELISA) (Fig. 3C). The release of another important chemokine not present in the screening panel, CXCL2, was evaluated in plasma, showing a similar pattern of expression as CINC1, confirming the beneficial effect of recAP administration (Fig. 3C). At the messenger RNA (mRNA) level, recAP reduced the intrahepatic expression of CXCL2, tumor necrosis factor alpha (TNFα) but not CCL2 (Supplementary Fig. 3).

#### Effect of recAP on AKI

LPS injection in BDL animals increased creatinine levels (p < 0.001) and reduced the renal blood flow (RBF) (p = 0.03). Both parameters were numerically but not significantly improved by recAP pretreatment (Supplementary Fig. 4C1,D2). In addition, although a trend to reduced urinary neutrophil gelatinase-associated lipocalin (NGAL) was observed in the recAP treated BDL + LPS group (n = 3) this was not statistically significant (Supplementary Fig. 4D1). Histologically, the kidneys from LPS-treated rats (Supplementary Fig. 4A3) showed evidence of congestion and presence of “ragged” proximal tubules, which was better preserved in the animals pre-treated with recAP (Supplementary Fig. 4A4). Bile staining in the tubules was associated with dilated proximal tubules and loss of brush border in the kidneys of the BDL rats (Supplementary Fig. 4A5). A marked worsening and distortion of the renal architecture was observed in the kidneys from the BDL + LPS animals (Supplementary Fig. 4A7), which was not prevented by recAP pre-treatment (Supplementary Fig. 4A8).

#### recAP reduces brain water content

BDL + LPS rats had a significant increase of serum ammonia levels compared with the BDL animals but this was not prevented by recAP (Fig. 4.2). BDL + LPS rats had numerically higher brain water compared to the BDL group (p = 0.093) (Fig. 4.1) and a significant reduction by recAP pre-treatment (p = 0.02).

**Figure 4:**
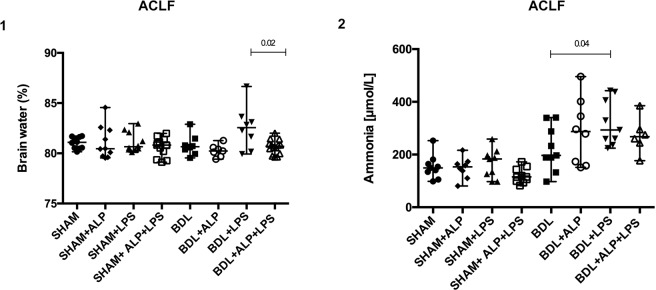
Relative brain water content and circulating ammonia levels in ACLF animals. The highly significant hyperammonaemia observed in the BDL + LPS group was not prevented by pre-administration of recAP (2) (ammonia levels available: n = 6 BDL + ALP + LPS, all indiviuals from the remaining groups). On the contrary, brain swelling, assessed by brain water measurement, was effectively prevented by recAP treatment prior to LPS infusion in the BDL animals (1) (brain water available: all individuals per group). Group comparisons for continuous variables were performed by using Mann-Whitney U test. A p-value ≤ 0.05 was considered significant. Only significant p-values were displayed in the figure.

#### recAP reduces LPS bioactivity but not endotoxin levels

**Figure 5:**
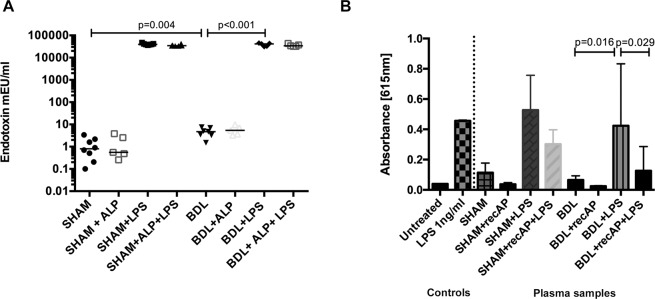
Treatment with recAP has no effect on total plasma endotoxin level but reduces bioactivity of circulating LPS in BDL animals. (**A**) Plasma endotoxin quantification by LAL assay (Sham n = 4, Sham + LPS n = 4, BDL n = 4, BDL + LPD n = 3, BDL + recAP n = 3, BDL + recAP + LPS n = 3) showed a significant increase in the endotoxin levels in the BDL compared with sham-operated rats (4.6 ± 0.8 vs. 2 ± 0.4; p = 0.004) and further increase after LPS infection (38889 ± 6322; p < 0.001). However, recAP pre-treatment had no effect on the total serum endotoxin levels in any of the recAP treated animals compared with control. (B) Using HEKBlue-hTLR4 reporter cells (Sham n = 8, Sham + recAP n = 5, Sham + LPS n = 7, Sham + recAP + LPS n = 6, BDL n = 7, BDL + recAP n = 5, BDL + LPS n = 5, BDL + recAP + LPS n = 5), plasma from the BDL + LPS animals showed a 13-fold increase in TLR4 transactivation compared to untreated controls. Similar transactivation was observed in positive controls (pure LPS, 1 ng/ml). Pre-treatment with recAP resulted in marked deactivation of circulating LPS as demonstrated by an 80% decrease in TLR4 transactivation induced by plasma of BDL + recAP + LPS animals, *p = 0.029. Group comparisons were performed by using Mann-Whitney U test between Sham-Sham/LPS, Sham/LPS-Sham/recAP/LPS, Sham-BDL, BDL-BDL/LPS and BDL/LPS-BDL/recAP/LPS. A p-value ≤ 0.05 was considered significant. Only significant p-values were displayed in the figure.

BDL rats had significantly higher endotoxin concentration compared with sham-operated controls (p = 0.004) and increased further with LPS administration compared to BDL (p < 0.001). Treatment with recAP had no effect on the serum endotoxin concentration (Fig. 5A). A marked activation of TLR4 signalling was observed in the HEK-Blue hTLR4 reporter cell line treated with plasma from the BDL + LPS animals, which was reduced by 80% in the recAP animals (p = 0.029) (Fig. 5B). Furthermore, adding recAP *in vitro* to human leukemic monocytes (THP-1) reduced their response to LPS after 24 hour of culture (Supplementary Fig. 5).

### Effects of LPS detoxification by recAP pre-treatment in a rat model of ALF

#### recAP does not prevent severity of liver injury in ALF

**Figure 6:**
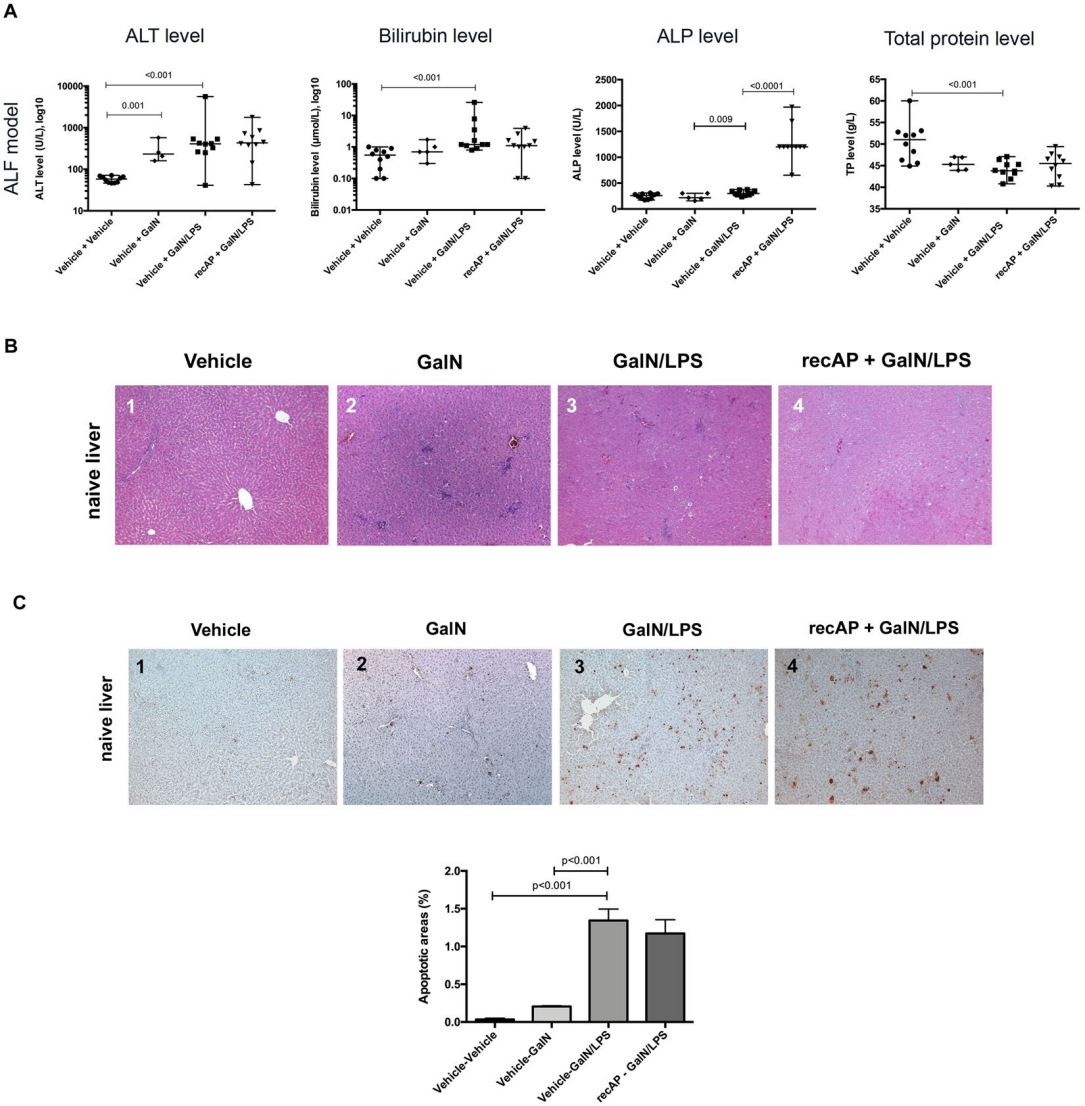
Liver biochemistry, H&E staining and apoptotic cell death (TUNEL staining) liver tissue in the ALF model. Plasma levels of ALT, Bilirubin, ALP and Total protein in the (**A**) ALF model induced by GalN/LPS. Parameters were measured in all animals per group. Only significant p-values are displayed in the graphs. Due to a high variance logarithmic scale was chosen to depict the results for ALT and Bilurbin. Details regarding the level of statistical significance are limited to the comparison between vehicle-vehicle and vehicle –GalN/LPS as well as the comparison between vehicle-GalN/LPS and recAP-GalN/LPS and are only displayed if significant. Liver tissue of all groups of the ALF model was stained with H&E (n = 4 per group) (**B**). RecAP treatment had no impact on histopathological changes in liver tissue. Injection of GalN in rats with naive liver induced histologically a liver injury with hepatocyte ballooning, necrosis and inflammatory cell infiltrations (**B2**). This finding was significantly enhanced when LPS was given in combination with GalN (**B3**). Pretreatment with recAP could no abrogate the damaging effect of GalN/LPS (**B4**) (Magnification X10). TUNEL staining of liver tissue was performed to detect apoptotic cell deathin rats from the ACLF model (**C**). GalN administration induced apoptotic cell death of hepatocytes throughout the whole liver tissue without predominance of periportal or central regions (**C2**). This finding was exagerated by combining GalN with LPS (**C3**) and remained unchanged after pretreatment with recAP (**C4)** (Magnification X20). Quantification of apoptotic areas was performed using ImageJ (n = 2 per group). Group comparisons for continuous variables were performed by using Mann-Whitney U test. A p-value ≤ 0.05 was considered significant. Only significant p-values were displayed in the figure.

Six hours after D-galactosamine (GalN) injection, ALT increased significantly (p = 0.001) (Fig. 6A, Supplementary Table 1). ALT (p < 0.001) and bilirubin (p < 0.001) further increased in the GalN + LPS group (Fig. 6A, Supplementary Table 1). Pre-treatment with recAP did not abrogate the severity of liver injury (Fig. 6A, Supplementary Table 1). Histopathologically, GalN-treated animals showed evidence of liver injury, further enhanced by LPS. Compared with the control animals, H&E staining of liver tissue showed hepatocyte ballooning, necrosis and inflammatory cell infiltration, which was unchanged in the animals pre-treated with recAP (Fig. 6B). On TUNEL staining, evidence of hepatocyte cell death was observed in the GalN treated animals and worsened further in the GalN + LPS animals, which remained unchanged in the recAP treated animals (Fig. 6C). Pre-treatment with recAP did not result in changes in chemo- and cytokine profiles in these ALF animals at both liver and plasma levels (Supplementary Fig. 6).

#### recAP does not reduce AKI or brain water in ALF

A non-statistically significant increase in creatinine levels was observed in animals treated with GalN and GalN/LPS compared to controls. In rats pre-treated with recAP, values were equivalent to GalN/LPS animals (Supplementary Fig. 4C2). Urea levels significantly increased in GalN and GalN/LPS treated animals (p = 0.004). However, pre-treatment with recAP did not improve urea levels (Supplementary Table 1).

GalN/LPS treated rats had similar brain water values compared to both, controls and recAP treated animals (Supplementary Fig. 7).

### Hepatocyte TLR4 expression in ALF and ACLF and effect of recAP

**Figure 7:**
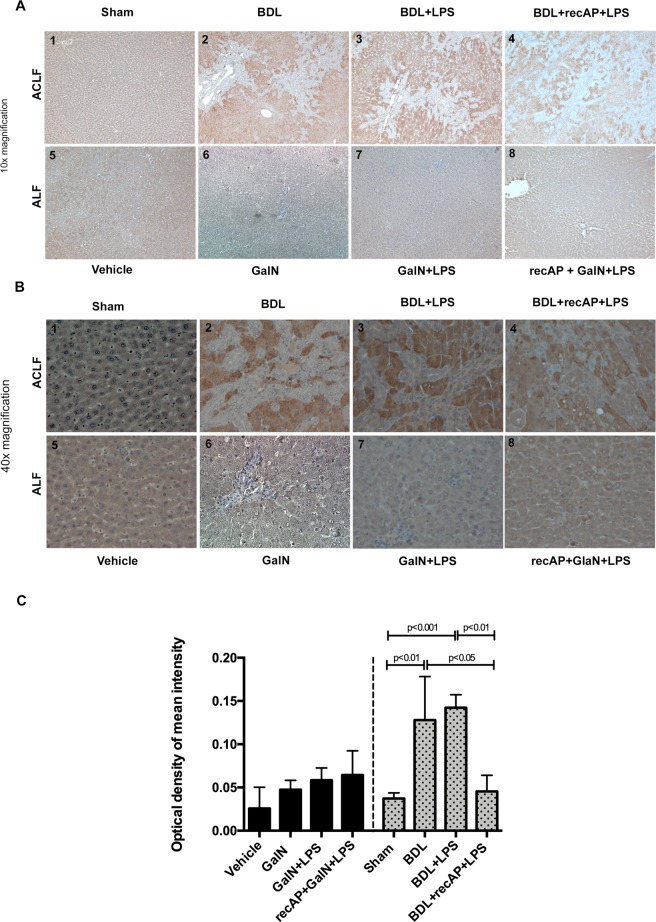
TLR4-expression in liver tissue (immunohistochemitry) from both rat models (**A** 10x magnification, **B** 40x magnification) (n = 4 per group). Liver tissue of control animals showed a low TLR4 expression (**A1,B1,A5,B5**). Once a chronic injury was induced by BDL there was a clear TLR4 upregulation in hepatocytes whereas regenerative zones did not express TLR4 (**A2,B2**). LPS injection did not alter this pattern significantly (**A3,B3**). Pre-treatment with recAP reduced TLR4 expression in hepatocytes (**A4,B4**). In the ALF model, neither GalN alone or with LPS (**A6,A7,B6,B7**) nor pretreatment with recAP did alter the TLR4 expression (**A8,B8**). DAB intensity quantification was performed on every single image by using Fiji/ImageJ (**C**). Group comparisons for continuous variables were performed using Mann-Whitney U test. A p-value ≤ 0.05 was considered significant. Only significant p-values were displayed in the figure.

Liver immunohistochemical analysis showed low basal expression of TLR4 in hepatocytes of control animals in both models. A marked increase in the TLR4 expression was observed after BDL and LPS infection. However, recAP pre-treatment significantly reduced the intensity of hepatocyte TLR4 expression (Fig. 7). This was also evident in BDL + recAP animals which were not treated with LPS injection (Supplementary Fig. 8). This occurred predominantly in the cytoplasm whereas diaminobenzidine (DAB) staining at the cell membranes remained unchanged, pointing towards a potential TLR4 re-distribution in response to recAP (Fig. 7). In contrast, inducing ALF by GalN/LPS did not upregulate TLR4 expression in hepatocytes, nor did recAP pre-treatment have an effect (Fig. 7). Hepatocyte origin of TLR4 expressing liver tissue cells was confirmed by co-staining for nuclear HNF4alpha and TLR4 (Supplementary Fig. 9).

## Discussion

The results of this study show that recAP, a drug that dephosphorylates and inactivates LPS, can prevent the development of ACLF but not of ALF despite both syndromes being precipitated by LPS administration in two well-established rodent models. The clinical benefits of recAP in ACLF were not only proven by diminishing ALT-levels, but also associated with a marked reduction in hepatic and systemic inflammation, improved intrahepatic apoptosis and moreover diminished brain edema. The mechanism of this discordant effect of recAP in ACLF and ALF may be related to the observation that TLR4 expression in hepatocytes was markedly elevated in the BDL animals and downregulated by recAP. However, in the GalN/LPS-induced ALF animals, the expression of TLR4 was low and remained unchanged after recAP. Therefore, the data shown here proposes potentially novel aspects of the interaction between the LPS-TLR4 axis and the liver. Chronic liver injury induced by BDL results in an increase in hepatocyte TLR4 expression and therefore, sensitizes the liver to LPS. The therapeutic potential of this observation is highlighted by the observation that downregulating TLR4 expression by administration of recAP reduces the susceptibility to LPS and prevents the development of ACLF. The fact that recAP acts through alteration of receptor expression in hepatocytes clearly supports the prophylactic use of recAP, in which the therapeutic capacity develops over time. In humans this might be equivalent to patients with acutely decompensated liver cirrhosis, who bear a certain risk to progress to ACLF.

Many lines of investigation indicate the importance of bacterial translocation and the resultant endotoxemia in mediating the complications of cirrhosis. Indeed, selective gut decontamination with norfloxacin and rifaximin are used for both prevention of complications of cirrhosis^25^ suggesting that LPS is indeed an important therapeutic target. Alkaline phosphatase subserves important protective roles including regulation of intestinal surface pH, absorption of lipids, detoxification of free nucleotides and bacterial LPS, attenuation of intestinal inflammation, and possible modulation of the gut microbiota^20^. The actions of alkaline phosphatase are due to its ability to reduce inflammation through dephosphorylation of lipopolysaccharide, which is a potent pathogen associated molecular pattern^21^. There is increasing evidence, that recAP mitigates sepsis associated AKI^23,26,27^. As recAP is taken up by the liver^28^, it is an attractive agent to try and minimize LPS-associated liver failure.

In order to test this hypothesis, we used two well-established animal models, the BDL/LPS and the GalN/LPS models recapitulating, ACLF and ALF respectively^29^. The most important observation of this study was that LPS administration to BDL animals resulted in exacerbation of hepatic and extrahepatic organ failures, which were abrogated by pre-treatment with recAP. Besides a reduced hepatocyte damage, as shown with ALT-levels and cell death markers, the mechanisms of the deleterious effect of LPS and the protection with recAP was the modulation of several cytokines and chemokines such as LIX and L-selectin, IL1a and CCL20, cytokine-induced neutrophil chemoattractant 1 (CINC1) and CxCL2. These are potent modulators of neutrophil homing^30^ and therefore might explain the reduced inflammatory cell infiltration observed in liver tissue of treated rats. Other organs such as the brain were similarly protected. The mechanism of the protection of the brain was not due to a reduction in ammonia but possibly due to the effects of recAP on systemic inflammation and deactivation of LPS, which exacerbates the deleterious effects of ammonia^31^. It would be necessary to investigate the effect of TLR4 inhibition on cerebral edema in this model in order to clarify whether LPS inactivation and thereby TLR4 signaling reduction mediates the effect. Additionally, circulating CINC1, has been shown to lead to increases in the permeability of the blood-brain barrier^32^. Although, the protective effect of recAP on the kidneys was not significant some improvements were noted. Serum creatinine and urinary NGAL levels were reduced in the recAP pre-treated animals but this reduction was not statistically significant. Histologically, the kidneys of the BDL animals showed worsening in inflammatory cell infiltration and tubular injury following LPS, which was not prevented by recAP. This observation of minimal protection of the kidneys is in contrast to the effect of recAP on sepsis-induced AKI^24^. It is possible that the reduced effectiveness of recAP is that the BDL animals may be due to concomitant bile acid nephropathy^33^.

Results from the THP1 cell lines, LAL assay and HEK TLR4 cell lines suggested inactivation of circulating LPS by recAP confirming its known mechanism. The concentration of plasma endotoxin was similar between BDL/LPS rats with and without recAP treatment, whereas reporter cells responded significantly less to the plasma from treated animals, indicating that the same amount of LPS exerted a lower stimulatory effect on TLR4 once treated with recAP. Alternatively, or in addition, the reduced effect on the reporter cell line may be due to reduced hepatocyte cell death and the consequent release of TLR4 ligands such as histones^34^.

The lack of any protective effect of recAP in GalN/LPS induced ALF is pathomechanistically intriguing. This discrepant result supports the hypothesis that it is not the administration of LPS itself that defined the severity of liver failure but it was possibly related to its binding sites, the main pathogen recognition receptor, TLR4^35,36^ which mediates the LPS effect on target cells. This hypothesis is supported by our previous observation that the protective effect of norfloxacin from LPS induced AKI was possibly due to reduced renal tubular TLR4 expression^37^. Moreover, Pickkers *et al*. recently published the results of a multicentre phase 2a/2b trial of recAP in sepsis-associated AKI. Whereas the primary endpoint of improvement of creatinine clearance after 7 days was not reached, recAP did improve the kidney function and survival after 28-day. This late onset treatment effect may suggest a therapeutic mechanism that requires long term recAP administration, which could alter receptor expression as we have seen in our models^38^.

In order to explore whether expression of TLR4 in the liver might explain differences in recAP treatment responses, we performed immunohistochemical staining of liver tissues from both models. In control animals, there was low basal TLR4 expression in hepatocytes. Once chronic liver injury was induced by BDL, TLR4 was markedly upregulated in hepatocytes. HNF4alpha co-staining with TLR4 confirmed the hepatocyte cell type carrying the TLR4 receptor. These data are supported by recently published observations that BDL in rats is associated with an increase in TLR4 expression in the liver^39,40^. This increase in TLR4 expression was in marked contrast to the GalN/LPS induced ALF, in which the TLR4 receptor was not upregulated in any of the groups. It has been hypothesised that GalN increases the sensitivity of the hepatocytes to LPS inducing TLR4-TNFα mediated liver injury^41,42^ and blocking TNFα effectively inhibited hepatocyte apoptosis in this model^43^. It is conceivable that it is not the hepatocytes but rather the immune cells, such as Kupffer cells^43,44^, mediate the liver injury in a TNFα -dependent manner.

The data presented above suggests that the upregulation of the TLR4 receptor in chronic liver injury is an important determinant of LPS-induced liver injury but the mechanism of how BDL results in upregulation of TLR4 receptors in the hepatocytes is unknown. It requires further work on the crosslink between LPS and hepatocyte TLR4 expression^45,46^.

In conclusion, this study shows that recAP is a potential novel therapy to prevent progression of acute decompensation of cirrhosis to ACLF but does not protect from development of ALF. These discrepant effects of LPS associated liver failure are potentially explained by the observation that hepatocyte TLR4 receptor expression is significantly increased in the BDL animals sensitizing these animals to the effect of LPS but not in animals with previously normal liver.

## Materials and Methods

All experiments were conducted in accordance with UK Home Office Animals (Scientific Procedures) Act 1986 (updated 2012) and the 3Rs principles and were embedded in a project licence (No.: 14378) approved by the UK Home Office. All animals were housed in an animal facility at 22 °C, in a 12 h light/12 h dark cycle with food and water ad libitum. Animals were randomly allocated to treatment groups.

### Animals

#### Model of acute-on-chronic liver failure

The rodent model of ACLF utilised in this study was as described previously^47^. Eight groups of adult male Sprague–Dawley rats (RRID:RGD_10395233) (n = 12 per group) were studied 4-weeks after either sham-operation or BDL. Animals were treated either with saline or recombinant human alkaline phosphatase (recAP; gifted AMPharma, Netherlands) − 1000 U/kg intraperitoneal on four consecutive days before LPS injection (days 25–28 after BDL). The last recAP injection was performed 3-hours before ACLF induction with LPS derived from Klebsiella pneumoniae (Sigma, UK) (0.3 mg/kg/hr, intravenously) or saline 0.9% (154 mmol sodium chloride (NaCl), vehicle). The animals in each group were terminated by exsanguination under general anaesthesia with isoflurane (Piramal Healthcare, USA) 3 h after LPS administration. The groups were as described in Supplementary Fig. 1. Hemodynamic measurements including PP and RBF was performed.

#### Model of acute liver failure

GalN (Sigma, UK) was injected intra-peritoneally in combination with LPS at a dose of 400 mg/kg (GalN) and 0.05 mg/kg (LPS) diluted in 0.9% (154 mmol) NaCl in adult male Sprague–Dawley rats. RecAP was given intra-peritoneal with a dose of 1000U/kg diluted in 0.9% (154 mmol) NaCl on 4 consecutive days prior to GalN injection. The last recAP injection was performed 3 -hours before GalN/LPS administration. NaCl 0.9% (154 mmol) was used as vehicle. Four groups were studied and animals sacrificed 6 hours after GalN/LPS or vehicle injection (Supplementary Fig. 1). All animals were alive at 6-hours after injection of vehicle or GalN or GalN/LPS.

### Sampling and storage

Blood was withdrawn from the carotid line or abdominal aorta as appropriate. Liver, brain and kidneys were snap frozen in liquid nitrogen. All samples including urine were stored at −80 °C. Organs were also harvested in formalin for histological assessment.

### Histopathological assessment and immunohistochemistry

Histology was performed using tissue fixed in 10% formalin for 24 h, dehydrated, and embedded in paraffin. For H&E histology sections (5 µm) were cut, stained using haematoxylin & eosin (Vector Laboratories, UK), and examined by independent pathologist blinded to group allocation. Specimens were imaged with a Carl Zeiss Axiovert 200 M microscope equipped with a Plan-Neofluar 10X/0.3 objective and an AxioCamMR2 camera system with Axiovision software (Carl Zeiss Inc., Germany). For immunhistochemistry, paraffin embedded liver tissue section of 5 µm were deparaffinised and rehydrated in Xylene and Ethanol, respectively. Following antigen retrieval with Tris-based antigen unmasking solution (Vector Laboratories Ltd, UK), tissues were blocked (Vectorstain Elite ABC-HRP kit; Vector Laboratories Ltd, UK), incubated with primary TLR4 (Diluted 1:200; Novus Biologicals, US, RRID:AB_2204994), HNF4α (Diluted 1:50; Novus Biologicals, US, RRID:AB_11023085) or RIP3 antibody (Dilution 1:500, Genetex, US, RRID:AB_2037881) and further processed with universal secondary antibody (Vectorstain Elite ABC-HRP kit; Vector Laboratories Ltd, UK), an avidin-biotin peroxidase complex technique using ABC kit (Vectorstain Elite ABC-HRP kit; Vector Laboratories Ltd, UK) and stained with DAB peroxidase substrate kit (Vector Laboratories Ltd, UK). Tissue was counterstained with haematoxylin (Vector Laboratories Ltd, UK), dehydrated and mounted with Vectamount permanent mounting medium (Vector Laboratories Ltd, UK). HNF4α and TLR4 co-staining was performed by using the ImmPRESS double staining polymer kit (Vector Laboratories Ltd, UK) containing the horseradish peroxidase (HRP) system with DAB/nickel to develop the brown/grey colour and the alkaline phosphatase system with ImmPACT Vector Red to develop the magenta colour (Vector Laboratories Ltd, UK). Terminal deoxynucleotidyl transferase biotin-dUTP nick end labeling (TUNEL) staining of deparaffinised and proteinase K-treated liver and kidney sections was performed using the *In-Situ* Cell Death Detection kit, POD (Roche, UK) as per manufacturer’s protocol.

### Biochemistry

Plasma samples were assessed using a Cobas Integra 400 multianalyser (Roche; UK).

### Kinetic turbidimetric limulus amebocyte lysate assay for endotoxin

Plasma endotoxin concentrations were measured using the Kinetic Turbidimetric Limulus Amebocyte Lysate Assay (Charles River Laboratories International Inc., MA, USA) according to the manufacturer’s instructions^48^.

### Determination of LPS activity using TLR4 reporter cells and human leukemic monocytes

The capacity of recAP to reduce LPS acitivity was assessed *in vitro* by measuring human leukemic monocytes (THP1) (Sigma-Aldrich, UK, RRID: CVCL_0006) IL1b secretion. 200 µL of a 250,000 cells/ml cell suspension were added to each well in 96 well tissue culture plates. Plates were incubated for 12 h at 37 °C. Cells were incubated with either cell culture medium (controls) or recAP containing cell culture medium with a concentration of 1.5 U/ml or 3U/ml for one hour. Thereafter, LPS was added to a final concentration of 100 ng/ml and 2000 ng/ml. Plates were incubated for a further 24 h at 37 °C and supernatant subsequently collected. IL1b concentration in undiluted supernatant was measured by IL1b ELISA (human IL1b DuoSet ELISA, R&D Systems Europe, UK) according to manufacturer recommendations. Cells were cultured and analysed in duplicates per condition.

Presence of bioactive LPS in plasma samples and its ability to activate TLR4 signalling pathway was assessed using ‘HEK-Blue hTLR4’ cells (InvivoGen, California, USA, RRID: CVCL_IM82)^49^. Briefly, 200 µL of a 100,000 cells/ml cell suspension were added to each well in 96well tissue culture plates. Plates were incubated for 48 h at 37 °C. 22 µL of plasma sample was added to every well. A positive (LPS) and negative control (culture medium) was included in each plate. Each plasma sample and controls were run in duplicate. Plates were incubated for a further 24 h. Secreted embryonic alkaline phosphatase (SEAP) activity was detected by addition of 20 µL supernatant to 180 µL alkaline phosphatase detection medium (QUANTi-Blue, InvivoGen, USA) in 96well plates, which were incubated at 37 °C for 1 h. SEAP activity was assessed by reading the absorbance at 620 nm. As some animal groups were treated with recAP (intestinal), which can interfere with SEAP quantification, all plasma samples used in the cell culture experiments were pre-heated for 25 minutes at 56 °C to inactivate the exogenous recAP (Supplementary Fig. 10A). LPS capacity to transactivate TLR4 signalling in HEK-BlueTLR4 cells was unaffected by heat-inactivated for 25 minutes (Supplementary Fig. 10B).

### Proteome Cytokine array in Liver Tissue

Liver homogenates were assessed using a proteome profiler array for rat cytokine (Rat cytokine Array, R&D Systems, UK) according to the manufacturer’s instructions. Liver homogenates of individuals of one group were pooled (100 µg protein per individual).

### Determination of Cytokines and Chemokines by ELISA

The levels of CINC-1 and Chemokine (C-X-C motif) ligand 2 (CXCL-2) chemokines (in liver homogenates and plasma of rats with ACLF, respectively) were determined using a commercial ELISA (R&D systems, UK) according to the manufacturer’s instructions. CINC-1 levels are expressed in absorbance units/μg of proteins.

### Methods for mRNA expression analysis (CXCL2, TNFa, CCL2)

Total RNA was extracted from liver tissues and then cleaned up by using QIAzol Lysis Reagent and RNeasy Kit (Qiagen, CA, USA), respectively, according to the manufacturer’s protocols. Samples were quantified and assessed using NanoDrop1000 System (Thermo Scientific, USA). 0.2 µg of RNA were retro-transcribed into cDNA using the QuantiTect Reverse Transcription Kit (Qiagen, CA, USA) and 1 µl of the cDNA sample was used to set up real-time PCR reactions using TaqMan gene expression assays for rat TNF-α, CXCL2, and CCL2 (Life technologies, CA, USA) and 7500 Fast Real-Time PCR System as per manufacturer’s protocol. Each sample was tested in duplicates. Target genes were normalized using UbC as endogenous control and their relative quantification was carried out with 2^−ΔΔCt^ method (where Ct represents the threshold cycle) using the samples form the sham animals as calibrator. (Details for the TaqMan assays used are detailed in Supplementary Table 2).

### Measurement of urinary biomarkers of acute kidney injury (AKI)

Urine samples from rats with ACLF were studied. Urinary levels of neutrophil gelatinase-associated lipocalin (NGAL) were assessed in duplicates using pre-coated 96 well ELISA plates (Abcam, Cambridge, UK).

### Brain Water determination

Immediately after termination, the whole brain was removed and brain tissue water content was determined using a dry weight technique as described previously^50^.

### Statistical analysis

Statistical analysis was performed using SPSS 22 software (SPSS Inc., Chicago; IL). Group comparisons for continuous variables were performed by using Mann-Whitney U test or Kruskal-Wallis-test and for categorical variables by using Chi-Square test. A p-value ≤ 0.05 was considered significant. Graphs were prepared with Prism (GraphPad, USA) and figures compiled in Adobe Photoshop (Adobe Systems, USA) (Supplementary Information). The sample size for the BDL experiment was calculated based on the results provided by Peters E *et al*.^23^ showing an intrahepatic interleukine 6 (IL6) reduction of about 40%. We hypothesised an effect size of 35% in our model with alpha error of 0.05 and power of 80% calculating a samples size of 12 animals per group. Based on the effect on ALT levels in the BDL experiment we expected conservatively an effect size of 20% for the GalN/LPS experiments. With an alpha error of 0.05 and power of 80% we calculated 10 animals per group. Animals in the GalN only group were treated to confirm an additional impact of LPS on the liver injury in this model.

## Supplementary information

Supplementary information.
