# Supplementary material for: Recombinant Alkaline Phosphatase Prevents Acute on Chronic Liver Failure
Source: Sci Rep. 2020 Jan 15;10:389. doi: 10.1038/s41598-019-57284-z (PMC6962206; doi:10.1038/s41598-019-57284-z)
Supplement: Supplementary file 1 — Supplementary information. [file 41598_2019_57284_MOESM1_ESM.docx]

**Recombinant Alkaline Phosphatase Prevents Acute on Chronic Liver Failure**

**Cornelius Engelmann^1,2*^; Danielle Adebayo^1*^**; Marc Oria^1^; Francesco De Chiara^1^; Simone Novelli^1,3^, Abeba Habtesion^1^; Nathan Davies^1^; Fausto Andreola^1#^; Rajiv Jalan^1#^

**Supplementary information**

**Supplementary Table 1: Biochemistry**

Plasma levels of Alanine Transferase (ALT), Bilirubin, Alkaline phosphatase (ALP), Total protein, Creatinine and Urea in all groups. Values are expressed as Mean± SEM. The levels of significance displayed here are limited to: a- comparison between sham and BDL, b- comparison between BDL and BDL/LPS, c- comparison between BDL/LPS and recAP-BDL/LPS, d – comparison between vehicle-vehicle and vehicle – Galactosamine, e- comparison between vehicle-vehicle and vehicle – Galactosamine/LPS, f –comparison between vehicle-Galactosamine/LPS and recAP-Galactosamine/LPS and only displayed if significant.

|  | **Sham** | **Sham +** **recAP** | **Sham +LPS** | **Sham+** **recAP +LPS** | **BDL** | **BDL+ recAP** | **BDL +LPS** | **BDL+** **recAP +LPS** | **Vehicle + Vehicle** | **Vehicle + GalN** | **Vehicle + GalN/LPS** | **recaAP + GalN/LPS** |
| --- | --- | --- | --- | --- | --- | --- | --- | --- | --- | --- | --- | --- |
| **ALT (IU/L)** | 46.5±3 | 74±9 | 81±11.3 | 62.5±10.4 | 78.9±7.8 (p=0.001^a^) | 69.5±8 | 139±15.8 (p=0.002^b^) | 104.6±10.5 (p=0.042^c^) | 57.7±9.7 | 299±186.5 (p=0.001^d^) | 355.1±155.7 (p<0.001^e^) | 574.1±483.8 |
| **Bilirubin (μm/L)** | 0.8±0.2 | 1.21±0.1 | 1.7±0.4 | 1.1±0.1 | 154±6 (p<0.001^a^) | 149±15 | 131±8 (p=0.036^b^) | 130±4 | 0.53±0.33 | 0.9±0.5 | 4.53±7.8 (p<0.001^e^) | 1.4±1.1 |
| **ALP (IU/L)** | 135±2 | 1630±689 | 165±21 | 1852±328 | 266±27 (p=0.016^a^) | 1652±554 | 352±59 | 2213±335 (p=0.001^c^) | 245.4±50.8 | 236.6±64.7 | 309.2±47.8 (p=0.009^e^) | 1272.1±347.7 (p<0.001^f^) |
| **Total Protein (g/L)** | 41.2±1.1 | 45.2±1.2 | 42.2±1.8 | 41.5±1.1 | 46.8±1.8 (p=0.021^a^) | 44.6±3.2 | 43.7±2 | 39.1±2.1 | 50.5±4.5 | 45.4±1.5 | 44.1±1.9 (p<0.001^e^) | 44.9±3 |
| **Creatinine (µmol/L)** | 29.1±1 | 39.9±2.6 | 39.7±2.9 | 32±2.7 | 28.7±1.1 | 30.1±1.3 | 47.8±3.1 (p<0.001^b^) | 39.1±3 | 17.4±1.6 | 16.5±2.5 | 19.8±4.5 | 20.1±2.9 |
| **Urea (mmol/L)** | 6.7±0.3 | 7.6±0.5 | 8.8±0.6 | 7.1±0.4 | 7.5±0.6 | 7.7±0.6 | 9.3±0.7 (p=0.027^b^) | 8±0.5 | 3.1±0.7 | 4.6±0.3 | 4.4±0.9 (p=0.004^d^) | 4.5±0.8 |

**Supplementary Table 2**

| Gene | Abbreviation | Assay ID (Company) |
| --- | --- | --- |
| Chemokine(C-C motif) ligand 2 | CCL2 | Rn00580555_m1 |
| Chemokine(C-X-C motif) ligand 2 | CXCL2 | Rn00586403_m1 |
| Tumor Necrosis Factor alpha | TNFa | Rn01525859_g1 |
| Ubiquitin C *(HK) | UbC | Rn01789812_g1 |

**Assays on demand from ThermoFisher Scientific (Applied Biosystems) for mRNA and miRNA quantification.** ***(HK): Housekeeping gene**

**Supplementary figures and figure legends**

**Supplementary figure 1: Design animal experiments**

**
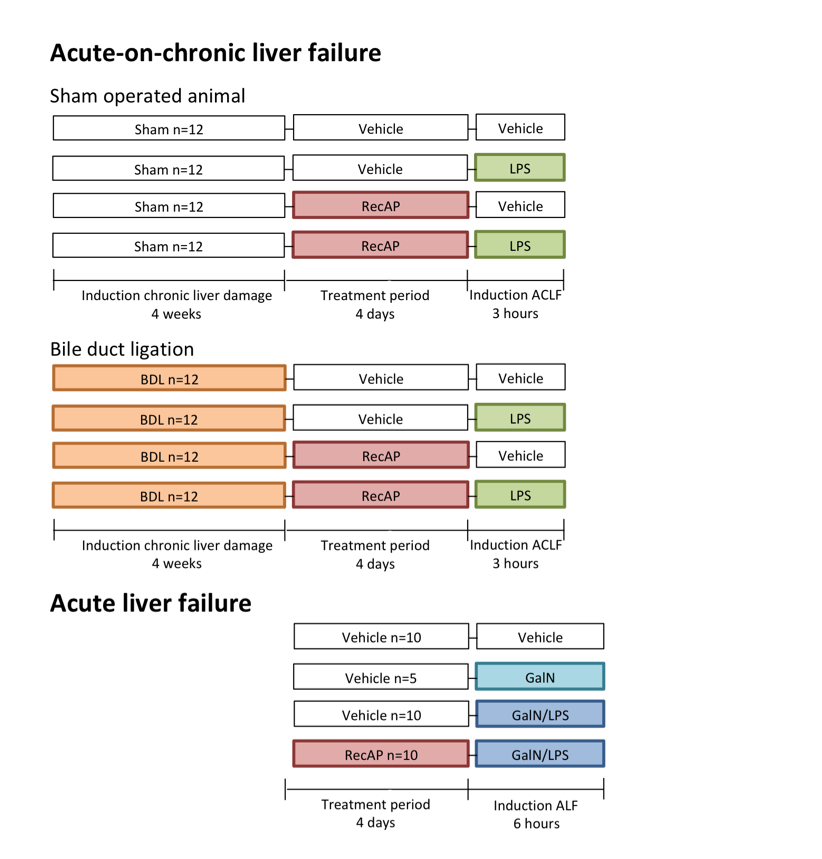
**

For the ACLF model chronic liver damage and advanced fibrosis was induced by bile duct ligation (BDL). Eight groups of adult male Sprague–Dawley rats (n= 12 per group) were studied 4-weeks after either sham-operation or BDL. LPS - Klebsiella pneumoniae lipopolysaccharide (Sigma, UK) (0.3mg/kg/hr, intravenously) was used as a second hit initiated organ damage mimicking ACLF. The animals in each group were terminated 3h after administration of LPS or saline 0.9% (154 mmol NaCL) under terminal anaesthesia. Acute liver failure was induced by intra-peritoneally injection of D-galactosamine either alone or in combination with LPS at a dose of 400mg/kg (GalN) and 0.05mg/kg (LPS) diluted in 0.9% (154mmol) NaCL in adult male Sprague–Dawley rats. Animals were treated either with saline or recAP – recombinant human alkaline phosphatase (Kindly gifted AMPharma, Netherlands) - 1000U/kg on four consecutive days before LPS injection. The last recAP injection was performed 3 hours before ACLF or ALF.

**Supplementary Figure 2: Portal pressure in ACLF model**

**
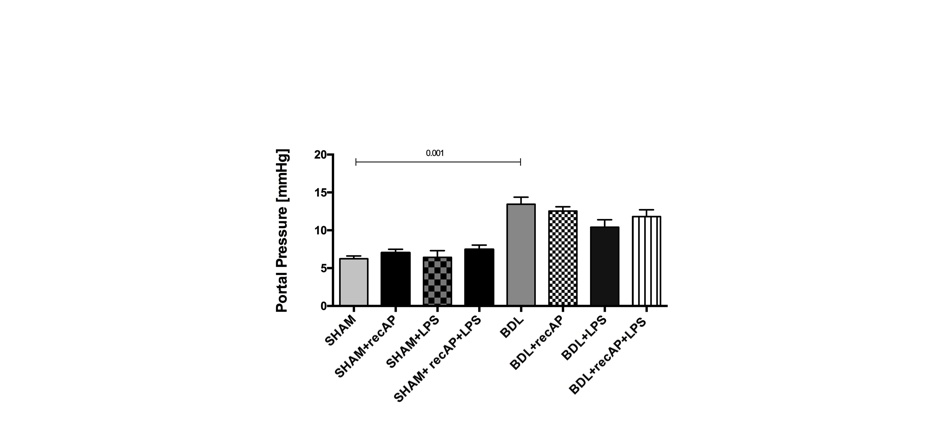
**

Rats with recAP pre-treatment had no changes in portal pressure (n=6 per group). A p-value ≤ 0.05 was considered significant. Only significant p-values were displayed in the figure.

**Supplementary Figure 3: mRNA expression of cytokines in liver tissue**


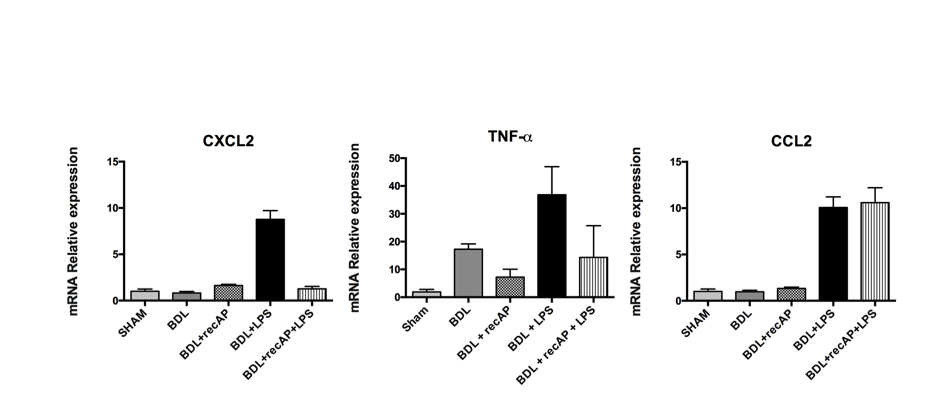
To further confirm the effect of recAP treatment in modulating circulating CXCL2 levels, we also assessed liver CXCL2 mRNA expression in the key treatment groups. CXCL2 mRNA shows a 8-fold up-regulation in the livers from BDL+LPS animals compared to sham controls; this up-regulation was significantly reduced in the BDL+recAP+LPS. Furthermore, we sought to quantify liver mRNA expression of TNFα, a commonly implicated cytokine in ACLF. A stepwise significant increase in TNFα mRNA expression was observed in the livers of the BDL and BDL+LPS rats, respectively, compared to sham controls. Pre-treatment of the BDL rats with recAP resulted in complete prevention of the further LPS-induced rise in TNFα mRNA expression. Treatment with recAP had no effect in preventing CCL2 mRNA up-regulation observed in liver of BDL+LPS animals.

**Supplementary Figure 4:** **Effect of recAP on kidney injury in ALF and ACLF**


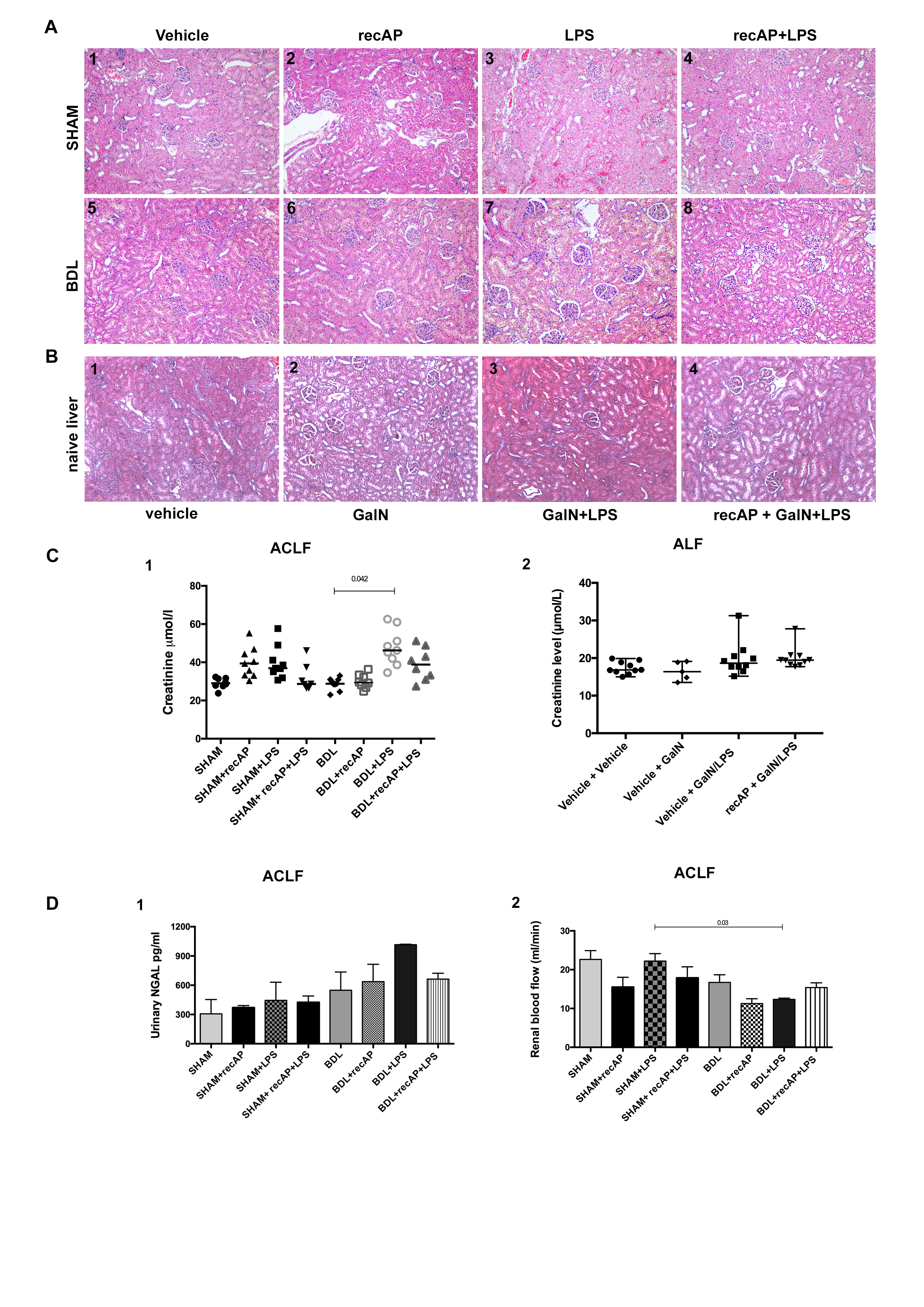


Effects of recAP pre-treatment on kidney histology in the (A, n=4 per group) ACLF model and ALF (B) model, (C) creatinine levels in both models and (D) renal blood flow and markers of renal injury in the ACLF model. A. Normal kidney histology in control (sham) rats (A1). Evidence of congestion and the proximal tubules appear “ragged” was observed in LPS-treated sham rats (A3). Better preservation of the renal histology was observed in sham+recAP+LPS (A4) compared with the sham+LPS. Evidence of brown pigmentation in the tubules associated with dilated proximal tubules and loss of brush border was observed in BDL (A5). Worsening of renal architecture shown in BDL+LPS was not improved in BDL+recAP+LPS (A7 and A8, respectively). recAP pre-treatment has no effect on renal histology in either the sham or the BDL recAP pre-treated animals (A2 and A6, respectively). Magnification x10. B. In kidneys of rats treated with Galactosamine alone or Galactosamine + LPS not such histomorphological damage was evident. C and D. Plasma levels of creatinine in the ACLF model were not altered in the recAP treated group compared to their respective control (C1). Renal blood flow could be obtained from the following groups: Sham n=8, BDL n=7, Sham+LPS n=5, BDL+LPS n=3, Sham+recAP n=4, BDL+recAP n=4, Sham+recAP+LPS n=8, BDL+recAP+LPS n=5. Renal blood flow was significantly reduced in the BDL+LPS group compared to the sham+LPS group (p<0.01) (D1). In addition, recAP had no effect on urinary NGAL levels (n=5 per group) in any of the treated groups compared to the control (BDL+LPS, n=3) (D2). Induction of liver injury in naive livers with Galactosamine/LPS tend to increase creatinine levels, but without reaching statistical significance (C2). Pretreatment with recAP did not alter creatinine levels. Group comparisons for continuous variables were performed by using Man-Whitney U test. A p-value ≤ 0.05 was considered statistically significant. Only significant p-values were displayed in the figure.

**Supplementary figure 5: THP-1 cell response to recAP related LPS inactivation**


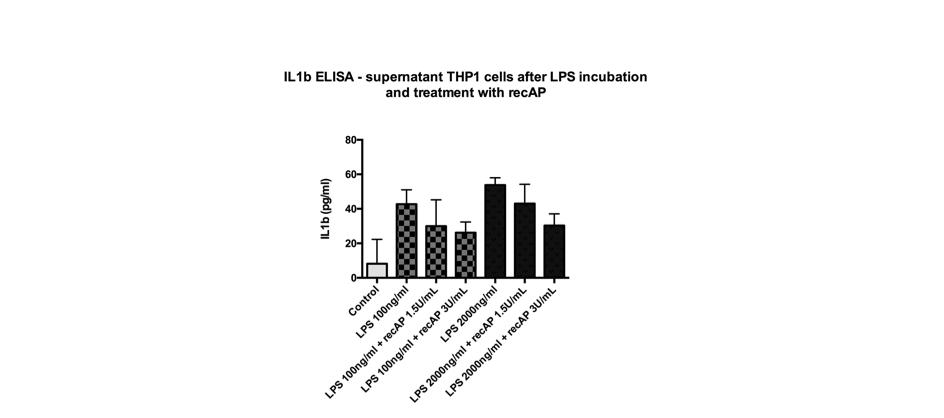


After 24 hours of incubation recAP effectively reduced response of THP-1 cells to LPS with a reduced IL1b secretion in a dose dependent manner. Cells were cultured analysed in duplicates per condition.

**Supplementary figure 6:** **Hepatic and plasmatic cytokine expression in ALF model**


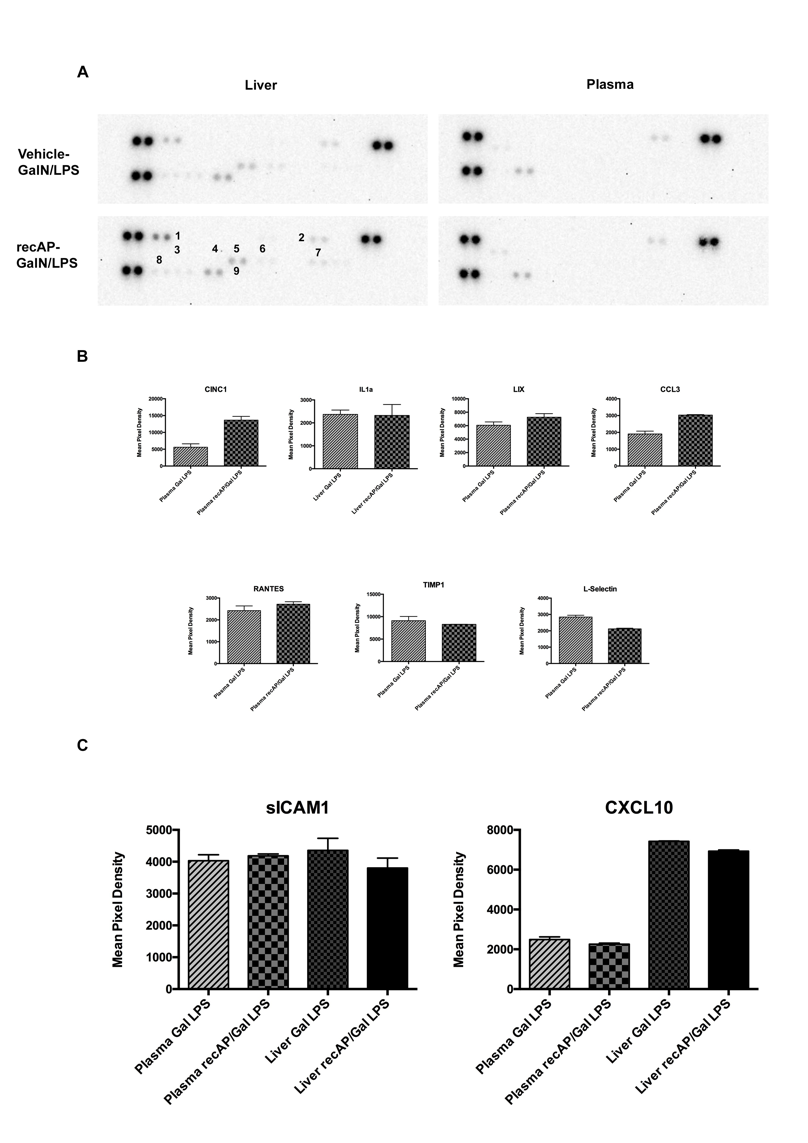


LPS detoxification with recALP in GalN/LPS induces ALF did not effect the intrahepatic and systemic cytokine release (A cytokine profiler, B densitometry liver tissue cytokine expression, C densitometry intrahepatic and plasmatic cytokine expression). Cytokine dot positions on the blots were as follows: dots 1 – CINC1; dots 2 – slCAM1; dots 3 – IL1a; dots 4 – CCL10; dots 5 – LIX; dots 6 – L-Selectin; dots 7 – CCL3; dots 8 – RANTES; dots 9 – TIMP-1. Pooled samples used for this analysis consisted of five individuals per group.

**Supplementary Figure 7:** **Relative brain water content in ALF animal model**


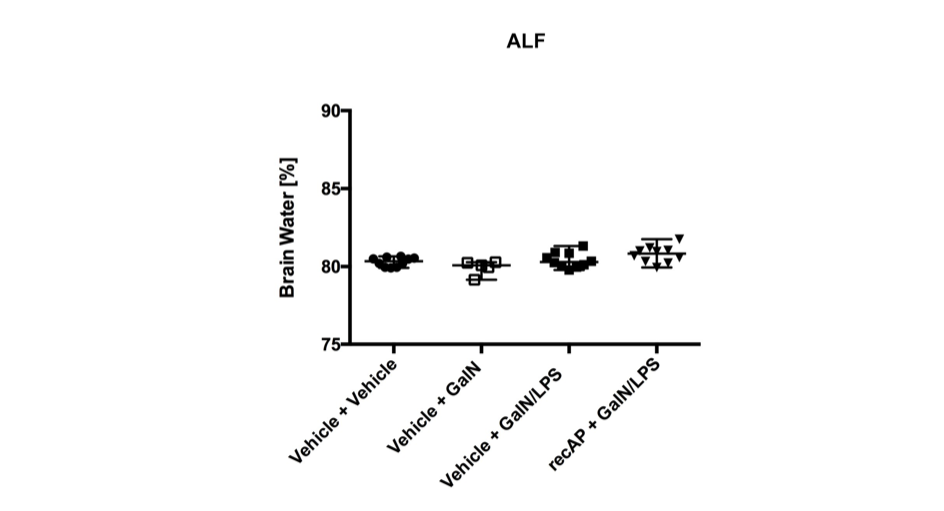


In the ALF model, neither did GalN/LPS increase the brain water content significantly nor did recAP show a therapeutic. Values were available for all individuals per group. A p-value ≤ 0.05 was considered significant. Only significant p-values were displayed.

**Supplementary figure 8: Liver TLR4 expression in bile duct ligated animals without LPS injection.**

**
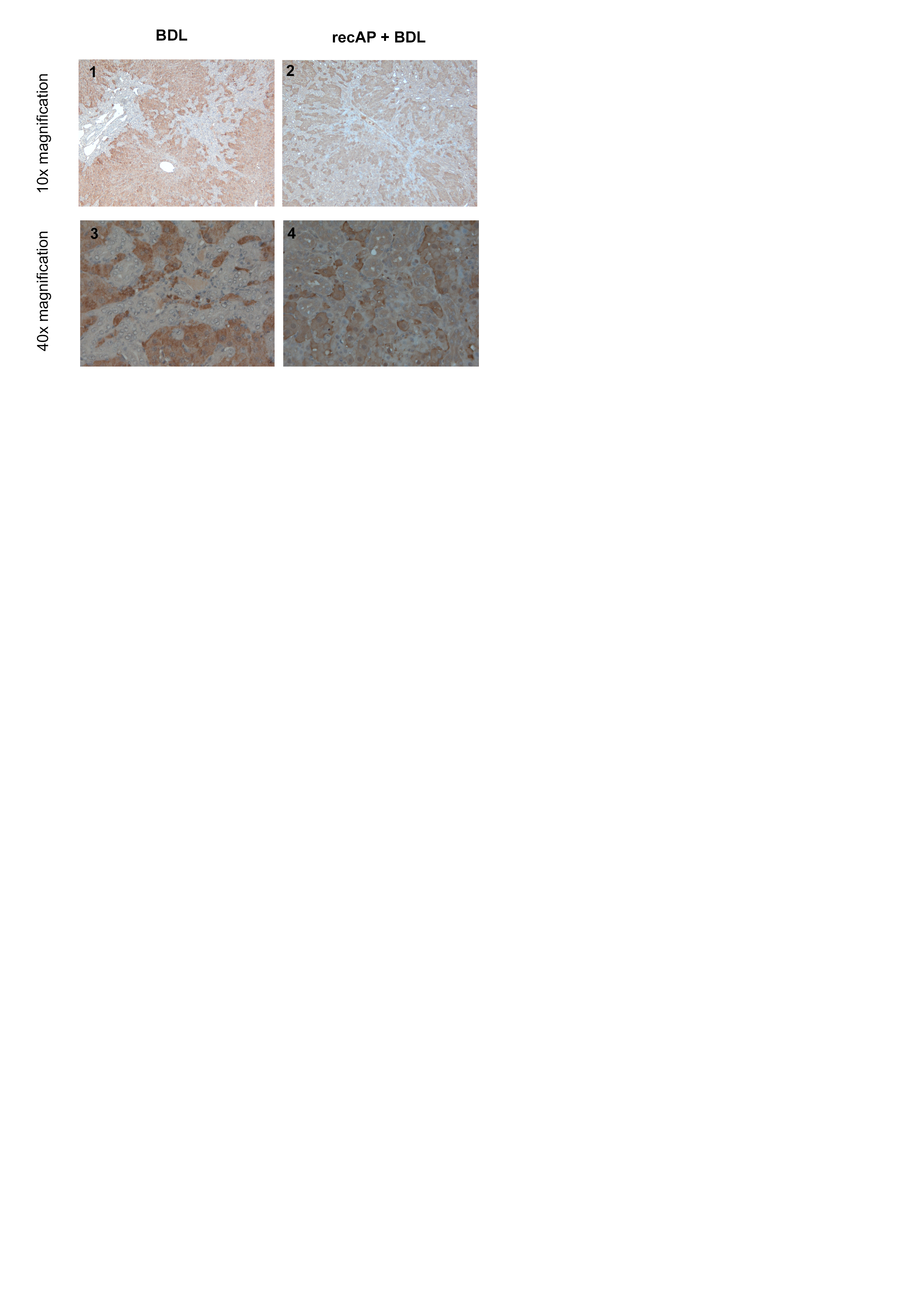
**

In BDL animals without LPS challenge treatment with recAP reduced the hepatocyte TLR4 expression (panel 1-4), equivalent to BDL animals with LPS injection (figure 7).

**Supplementary figure 9: Co-staining of hepatocytes for HNF4α and TLR4**


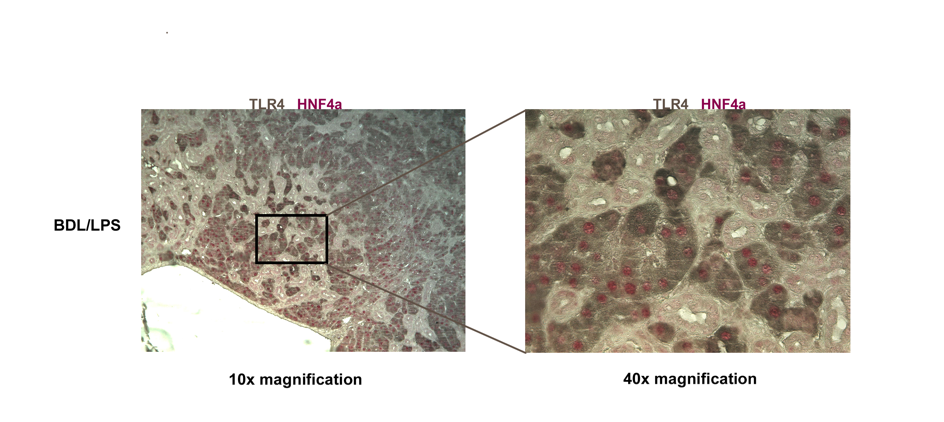


Immunohistochemical co-staining of liver tissue with HNF4α antibody (hepatocyte nucleus staining, magenta) and TLR4 (cytoplasmatic staining, brown/grey) showed that areas with high TLR4 expression are of hepatocyte origin.

**Supplementary Figure 10: Heath-inactivation of plasma from recAP-treated groups effectively eliminated the exogenous residual exogenous recAP**


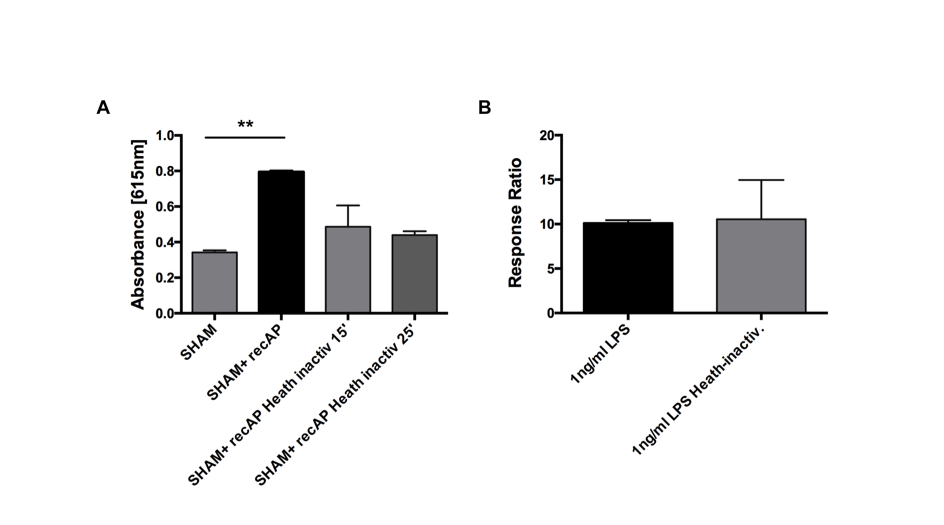


25 minutes of heat inactivation effectively deactivate the residual exogenous recAP (Panel A) without altering the LPS ability to transactivate TLR4 signalling in HEK-Blue TLR4 cells (Panel B). A p-value ≤ 0.05 was considered significant. Only significant p-values were displayed (** p<0.01)

**Supplementary Figure 11: Effect of recAP on cell death (TUNEL) in liver tissue of sham treated animals**


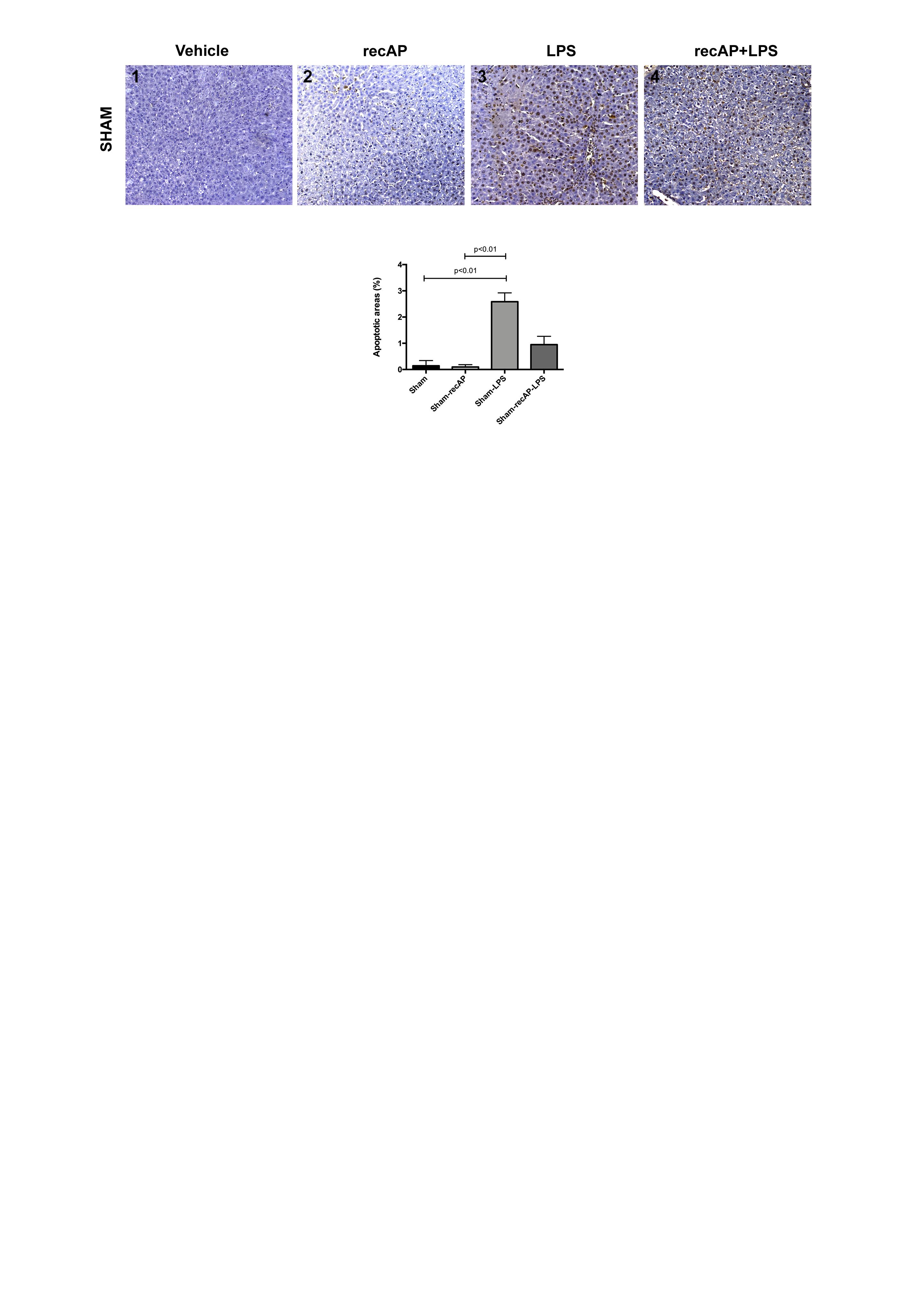


TUNEL staining of liver tissue was performed to detect apoptotic cell death. There were no signs of apoptosis in normal (sham), sham+ recAP-treated animals (1 and 2, respectively). LPS treatment alone induced apoptosis and recAP pre-treatment (recAP +LPS) attenuated the response (3 and 4, respectively).

Quantification of apoptotic areas was performed using ImageJ (Sham n=5; Sham+recAP n=4; Sham+LPS, Sham+recAP+LPS n=7). Group comparisons for continuous variables were performed by using Mann-Whitney U test. A p-value ≤ 0.05 was considered significant.
